# Cough and cold medicine prescription rates can be significantly reduced by active intervention

**DOI:** 10.1007/s00431-021-04344-0

**Published:** 2021-12-15

**Authors:** Péter Csonka, Paula Heikkilä, Sonja Koskela, Sauli Palmu, Noora Lajunen, Sari Riihijärvi, Heini Huhtala, Matti Korppi

**Affiliations:** 1 Tampere Center for Child, Adolescent and Maternal Health Research, Faculty of Medicine and Health Technology, Tampere University and Tampere University Hospital, Arvo Ylpönkatu 34 (ARVO B235), 33014 Tampere, Finland; 2 Terveystalo Healthcare, Tampere, Finland; 3 Terveystalo Healthcare, Helsinki, Finland; 4 Faculty of Social Sciences, Tampere University, Tampere, Finland

**Keywords:** Cough medicine, Expectorants, Clinical practice, Guideline implementation, Healthcare practice, Quality assessment, Intervention

## Abstract

Our aim was to construct and test an intervention programme to eradicate cough and cold medicine (CCM) prescriptions for children treated in a nationwide healthcare service company. The study was carried out in the largest private healthcare service company in Finland with a centralised electronic health record system allowing for real-time, doctor-specific practice monitoring. The step-by-step intervention consisted of company-level dissemination of educational materials to doctors and families, educational staff meetings, continuous monitoring of prescriptions, and targeted feedback. Outreach visits were held in noncompliant units. Finally, those physicians who most often prescribed CCM were directly contacted. During the intervention period (2017–2020), there were more than one million paediatric visits. Prescriptions of CCMs to children were completely eradicated in 41% of units and the total number of CCM prescriptions decreased from 6738 to 744 (89%). During the fourth intervention year, CCMs containing opioid derivatives were prescribed for only 0.2% of children aged < 2 years. The decrease in prescriptions was greatest in general practitioners (5.2 to 1.1%). In paediatricians, the prescription rates decreased from 1.5 to 0.2%. The annual costs of CCMs decreased from €183,996 to €18,899 (89.7%). For the intervention, the developers used 343 h and the attended doctors used 684 h of work time during the 4-year intervention. The costs used for developing, implementing, reporting, evaluating, communicating, and data managing formed approximately 11% of total intervention costs.

*Conclusion*: The study showed that a nationwide systematic intervention to change cough medicine prescription practices is feasible and requires only modest financial investments.

**Table:** 

**What is Known:** *• Cough and cold medicines (CCM) are not effective or safe, especially for children aged 6 years.* *• Although the use of CCMs has been declining, caregivers continue to administer CCMs to children, and some physicians still prescribe them even for preschool children.*	
**What is New:** *• A nationwide systematic intervention can significantly and cost effectively change CCM prescription habits of paediatricians, general practitioners, and other specialists.* *• Electronic health records provide additional tools for operative guideline implementation and real-time quality monitoring, including recommendations of useless or harmful treatments.*

**Supplementary information:**

The online version contains supplementary material available at 10.1007/s00431-021-04344-0.

## Introduction

Cough belongs to the common symptoms for which medical advice is sought both in primary care and in specialist practice. In children, cough is mostly related to acute respiratory infection [1], post-viral epithelial damage, or increased cough receptor sensitivity [2], and serious diseases are very rare. Irrespective of the underlying condition, cough and cold medicines (CCM) as a choice of therapy for children are highly discouraged by most doctors and researchers [3–8]. CCMs have not been proven to be safe or effective, especially for children aged < 6 years [9–12].

Although the use of CCMs has been declining [13], caregivers continue to administer CCMs to children, and some physicians still prescribe them even for preschool children [14]. Parental concerns about their children’s cough can be overwhelming due to disturbed sleep or fear of severe disease [15]. Caregiver education is the mainstay of management of cough, and recommendations by physicians no doubt influence parental attitudes to CCMs [16].

Treatment guidelines decrease the gap between research and practice and, thus, reduce inadequate treatments and inappropriate variability in practice [17]. Unfortunately, guidelines are not applied in clinical practice for several reasons. These include barriers related to physicians’ knowledge (e.g. lack of awareness), barriers that affect physicians’ attitudes (e.g. lack of agreement), and patient-related barriers [18]. Nevertheless, evidence regarding the ways how to translate the identified barriers into tailored interventions is scarce [19].

The Finnish Current Care Guidelines on lower respiratory tract infections (LRTI) in children published in 2014 did not recommend any use of CCMs [20]. However, the rate of CCM prescription was unaffected by these guidelines. In a recent Finnish study, cough suppressants were prescribed to 8.5% of children before and to 9.7% after the publication of the guidelines [21].

Our primary aim was to construct and test an intervention tool to eradicate CCM prescriptions for children < 18 years of age treated in a private healthcare service company with approximately 300 clinics across Finland. We also evaluated the costs for the company and savings for the customers resulting from the intervention based on reinforcing guidelines. There are no previously published interventional studies focusing on CCM recommendations and prescriptions.

## Methods

### Setting

This prospective intervention study took place in Terveystalo, the largest private healthcare service company in Finland, which offers primary and secondary healthcare services for corporate and private customers as well as for the public sector. The nationwide network includes approximately 300 clinics across the country and a total of 13,000 medical doctors with over 50 specialties. Annually, Terveystalo serves more than 1.2 million individual customers. All diagnoses and prescriptions are recorded online into a centralised electronic health record (EHR) system. See more detailed description of the EHR and monitoring system in Appendix 1.

### Subjects and collected data

EHR data were collected on all children < 18 years old treated in any of the Terveystalo units between January 2014 and December 2020. Collected data included visit location, visit date, age at the visit, diagnosis code, all medications prescribed at the visit, the doctor’s identification code, and the doctor’s specialty. Prescribed CCMs were identified by the Anatomical Therapeutic Chemical (ATC) classification system codes (Appendix 2). All the above data were entered into the EHR as obligatory information. Predefined stratified analyses were performed for age groups by diagnostic classification of cough, upper respiratory tract infection (URTI), and LRTI (see diagnosis codes in Appendix 3).

### Intervention

First, the rate of CCM prescription was assessed in December 2016. During 2017, we regularly disseminated the document of the National Finnish Current Care Guidelines for lower respiratory infections in children [20], including reminders about guidelines for physicians via e-mail and in the company’s intranet. In January 2018, the rate of CCM prescriptions was re-evaluated, and in February 2018, the plans for the active intervention were completed (Appendices 4 and 5).

The active intervention was carried out in three steps and lasted from March 2018 to December 2020 (Appendix 5). In the first step (March–December 2018), we organised educational meetings supplemented with general, company-level dissemination of practical written materials. The motivation, feedback, and education programmes were continued throughout the entire intervention period. In the second step (March–June 2019), we performed outreach visits and organised focus meetings in identified noncompliant units. The second step also included direct feedback and reminders to those physicians who continued to prescribe CCMs for children. Finally, in the third step (September 2019–December 2020), those 50 physicians who most actively prescribed CMMs were directly contacted by personalised letters followed by phone calls if needed. The principal goal of the intervention was to reduce CCM prescriptions to zero. Supporting materials for the intervention are described in Appendix 6.

### Cost data

In Finland, the current prices of medicines are the same in all pharmacies, and prices are determined by the reimbursement of wholesale trade, taxes, and sales margins defined beforehand. The evaluation of CCM costs was carried out using the current prices in 2021 found from the Pharmaceutical Information Centre, Finland. The price of each product was then multiplied by the number of prescriptions in each year, with the presumption that every prescribed CCM was purchased. All costs, including prices of medicines and, e.g. salaries, were expressed in euros (€) at the 2021 level. See Appendix 7 for more detailed description of cost estimation.

### Statistical analysis

All doctoral visits during the study period were included in the analysis; therefore, power analyses or formal sample size calculations were not needed. The 95% confidence intervals (95% CI) for proportions were calculated by the Wald method (Stata 16.1, TX, USA). The software IBM SPSS Statistics for Windows, version 26 (IBM Corp, NY, USA), was used for the data analysis.

This was a quality assessment and development project. All data was coded, and patients were not contacted. According to the Finnish law, approval from the ethics committee was not required. The chief medical officer of Terveystalo gave the permission for the study.

## Results

Our data included all 1,629,187 paediatric visits that took place in any of the units of Terveystalo from 1.1.2014 to 31.12.2020. The age was < 2 years in 12.2%, 2–4.9 years in 26.8%, 5–11.9 years in 31.1%, and 12–18 years in 29% of children. Most visits were to GPs (31.9%); paediatricians (PED 29.7%); or ear, nose, and throat specialists (ENT 12.4%).

**Fig. 1:**
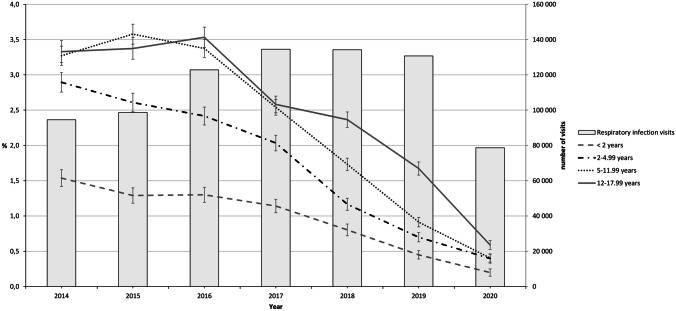
Proportion (%) of children receiving cough and cold medicine prescriptions each year in four age groups. Error bars indicate 95% confidence intervals (95% CI). Lines represent age groups. Bars represent numbers of visits due to respiratory infections. Total number of visits during 2014–2020: *n* = 1,629,187

**Fig. 2:**
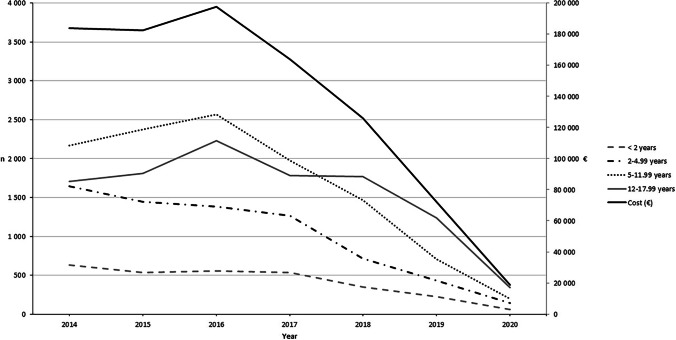
Number of cough and cold medicine prescriptions each year by age group (*n* = 32,264). The solid black line represents the total annual cost for all age groups in euros

The proportion of children receiving CCM prescriptions decreased significantly from 2016 to 2020 in all age groups, and the change was more pronounced during the active intervention in 2018–2020 (Fig. 1). The number of visits for respiratory infections did not decrease substantially between 2016 and 2019. However, there were significantly less respiratory infection-related visits in 2020, coinciding with the COVID-19 pandemic (Fig. 1). Before the active intervention, children aged > 5 years were more likely to receive CCM prescriptions compared to children aged < 5 years. By the end of the study, this age difference became significantly narrower. The total number of CCM prescriptions decreased from 6738 during 2016 to 744 during 2020 (89%), and the annual total costs of CCMs decreased from €183,996 in 2014 to €18,899 in 2020 (89.7%) (Fig. 2). Before the intervention, there were 73 units where physicians prescribed CCMs for children, compared to 43 units during the last year of the intervention. This means that 41.1% of the units reached the targeted zero prescription rate.

**Fig. 3:**
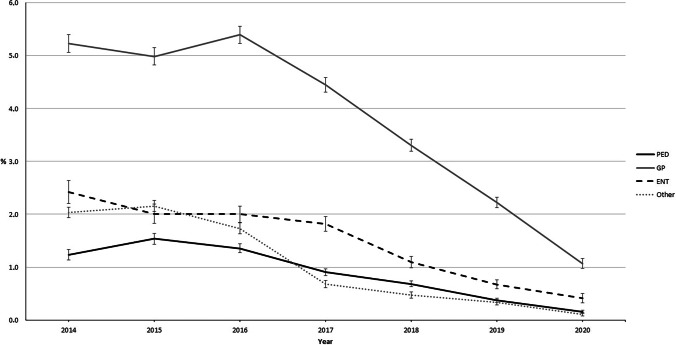
Proportion (%) of children receiving cough and cold medicine prescriptions by specialty each year. Error bars indicate 95% confidence intervals (95% CI). PED, paediatrician; GP, general practitioner; ENT, ear, nose, and throat specialist; Other, all other specialties. Total number of visits during 2014–2020: *n* = 1,629,187

CCMs were more likely to be recommended by GPs than by PEDs, ENTs, or other specialists (Fig. 3). The most notable reduction in prescription activity was seen among GPs (from 5.4% in 2016 to 1.1% in 2020).

**Fig. 4:**
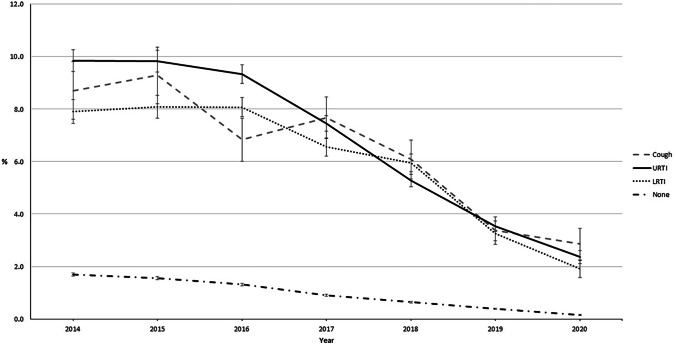
Proportion (%) of children within each diagnosis group receiving cough and cold medicine prescriptions. URTI, upper respiratory tract infection; LRTI, lower respiratory tract infection; None, no cough, URTI, or LRTI. Error bars indicate 95% confidence intervals (95% CI)

CCMs were also prescribed at visits that were due to causes other than cough, URTI, or LRTI, but the rate of CCM prescriptions was much higher when primarily related to visits for respiratory tract symptoms (Fig. 4).

The number of prescriptions declined for all CCM groups, such as suppressant and expectorant combinations, suppressants without combinations, and expectorants without combinations (Figure S1). Among children < 2 years of age, the prescription rate of CCMs containing opioid derivatives decreased from 1.1% in 2016 to 0.2% in 2020.

For the intervention, the developers used a total of 343 h of work time (including communicating and data management) during the 4-year intervention period, and the attending doctors used 684 h, respectively (Table 1). The intervention costs were €159,707 in total (Table 1), consisting mainly of participating the professional meetings and reading the material. The costs used for developing, implementing, reporting, evaluating, communicating, and data managing formed approximately 11% of total intervention costs.

**Table 1:** The costs of intervention were evaluated as work time multiplied by the mean salaries of intervention developers or missed mean invoicing of participants during December 2016 and February 2021

	**Time (hours)**	**Costs (€)**
The intervention’s development, implementation, reporting, and evaluation*	307	15,884
The communication offices’ work	3	63
The data management	33	863
Participants^†^	684	142,897
**Total**	**1,027**	**159,707**

^*^Developers’ work time was divided between the years 2016 and 2021

^†^Each participant used less than an hour of work time during intervention; each one read material and emails, and some of them participated in the meetings. Only a minority of them received personal guidance via phone calls or emails. Fifteen participants, who were chief physicians, used one more hour of work time compared to others

## Discussion

The main result of the present study was that a systematic 4-year intervention consisting of general and targeted releases of information and personalised feedback to doctors had a major impact on lessening CCM prescriptions for children in a large nationwide healthcare company with an average of 260,000 annual paediatric visits. The goal of the intervention was to completely eradicate the prescriptions of cough and cold medicines to children, and that goal was reached in 41% of the units. The goal was nearly reached concerning children < 2 years of age, since doctors prescribed CCMs to only 0.2% of them in 2020, in the fourth intervention year. In older children, the prescription rate was 0.4–0.6%, which means an over 80% decrease compared to 2014–2016 before the intervention. In paediatricians, the CCM prescription rate decreased from 1.3% in 2016 to 0.1% in 2020, and in GPs from 5.3 to 1.1%, respectively.

The goal of our intervention that CCMs should not be prescribed for children comes from the national and international guidelines [20]. The US Food and Drug Administration advised that children < 2 years of age should not at all use CCMs [5]. The American Academy of Pediatrics (AAP) and Health Canada recommended that children < 6 years of age should not use CCMs [3, 6]. The worldwide Choosing Wisely initiative includes avoiding of CCMs in treating children. In the Finnish Current Care Guidelines, there is a Smart to Avoid section (Choosing Wisely Finland) with similar recommendations. In the USA, the medical industry has labelled CCMs only for children > 4 years [4]. There is no evidence that CCMs are effective at any age, but the safety worries concern especially young children. In Finland, CCMs belong to the over-the-counter drug category, which highlights public release of information.

Among the most vulnerable, < 2-year-old children, the number of opioid-derivative prescriptions was 487 in 2016. Although the prescriptions decreased significantly, there were still 52 opioid-derivative prescriptions made for them in 2020. This is against recommendations. In addition, doctors should actively inform parents and guardians that CCMs are not useful and may be harmful especially for young children.

A successful introduction of guidelines involves three steps: development, dissemination, and implementation [22]. Evidence-based guidelines, which are usually national like the Finnish Current Care Guidelines, should be formatted to local guidelines in healthcare organisations, such as hospitals, healthcare centres, and clinics of healthcare companies. The success of implementation depends on the number and homogeneity of the doctors in question. In Finland, an example of successful guideline implementation was the recommendation not to use bronchodilators for infants with bronchiolitis [23]. Those guidelines were targeted to a small number of paediatricians who were responsible for bronchiolitis treatment in hospitals. When the problem is common, such as cough, and concerns large and heterogeneous groups of doctors, more powerful and versatile interventions like the present are needed.

A recent study from Australia and New Zealand included hospitals which treated more than 8000 infants with bronchiolitis during three seasons. Compliance with recommendations was significantly higher (85.1%) in intervention hospitals compared to 73% in control hospitals. The conclusion was that targeted interventions, including site-based clinical leads, stakeholder meetings, train-the-trainer workshops, targeted educational delivery, other educational and promotional materials, audits, and feedback systems, were more effective than passive dissemination of evidence-based guidelines in improving the treatment [24].

Likewise, in an outpatient study from the USA, personalised audits and feedback interventions were able to steer antibiotic prescribing practices for 316 children with community-acquired pneumonia, which is a rather common but however limited problem [25]. The intervention group had fewer (5.9%) non-guideline-concordant antibiotics prescribed, compared to 21.4% in controls.

An inpatient study from the USA showed that a local-level quality improvement initiative for decreasing the use of codeine-based analgesics could be implemented successfully with low costs [26]. The campaign consisted primarily of a widely distributed reference card in a 175-bed tertiary care hospital. As a result, the resident physicians showed a significant decrease from 13.5 to 5.4% in the rate of codeine prescriptions [26]. A recent study from Finland, which is the only paediatric outpatient study published thus far on the impact of guidelines on the use of cough medicine, did not show any benefits in 1661 children [21], when the guideline release was the only intervention.

Only a few paediatric studies have compared both treatment practices and the costs before and after guideline implementation. In the USA, time series analysis including 2929 infants with bronchiolitis treated in an emergency department revealed a reduction of both unnecessary treatments and mean costs per patient of $197 after guideline implementation, which means 17% saving [27]. Another retrospective cohort study from the USA including 267 children with bronchiolitis found that the highest adherence to bronchiolitis clinical pathways was associated with the lowest costs both in the emergency department (savings of $84 per patient, 12%) and in the hospital (savings of $1296 per patient, 23%) [28].

This present study, however, estimated not only direct cost savings for patients relating to reduced use of CCMs but also the costs of the intervention for the company. The total costs of the intervention (i.e. the investment done by Terveystalo) were approximately €160,000. The saved costs were higher (€346,946) when the years 2018–2020 were compared to the years 2014–2016. It is noteworthy that the investment was covered by the service provider, but the families benefitted from the cost savings. Thus, our results show that an intervention like this can be nationwide, successful, and cost-effective at the general level of healthcare, even in common diseases that are treated by a heterogeneous group of physicians.

The main strengths of the present study are the 4-year systematic intervention, a centralised collection of digitalised data on CCM prescriptions for 2 years before and 4 years during the intervention, and the great number (over 1.6 million) of paediatric visits, which included all eligible cases and covered different areas of the country.

Our intervention was not randomised or controlled. Even a cluster randomisation was not conceivable because significant cluster contamination would have been inevitable. Terveystalo has a centralised medical leadership, unified EHR system, quality surveillance, and operational decision-making processes. In addition, all company-related information and instructions are rapidly distributed via a common intranet. We evaluated CCM prescriptions to children, but the caregivers may buy most CCMs without prescriptions. Therefore, the goal of the study was not only to reduce prescriptions but also to educate the parents not to give CCMs to their children.

The COVID-19 pandemic started at the end of 2019 and was ongoing during the last year of our study. The common experience has been that, because of social restrictions and hygiene recommendations, the circulation of all respiratory viruses was less during than before the pandemic [29], and this has decreased doctoral visits and may have further reduced the overall prescriptions of CCM. However, CCM prescriptions showed significant reductions already before the pandemic.

Only limited data are available about prescription practices in Finland, and therefore, it is not possible to compare prescription habits and trends between different health service providers. CCM wholesale statistics are provided by the Finnish Medicines Agency (Fimea) but only as combined data including purchases for both adults and children, and purchases with and without prescriptions. According to Fimea, the defined daily doses per 1000 inhabitants and per day decreased by 34% from 2017 to 2020. During the same period, the CCM prescription rate decreased by 89% in our study.

In conclusion, this study confirmed that a nationwide systematic intervention to change cough and cold medicine prescriptions is feasible and requires only modest financial investments. Electronic health records provide tools for real-time quality monitoring and operative guideline implementation.

## Supplementary Information

Below is the link to the electronic supplementary material.

Supplementary file1 (PDF 169 KB)

Supplementary file2 (PDF 86 KB)

Supplementary file3 (PDF 87 KB)

Supplementary file4 (PDF 78 KB)

Supplementary file5 (PDF 107 KB)

Supplementary file6 (PDF 131 KB)

Supplementary file7 (PDF 80 KB)

Supplementary file8 (PDF 81 KB)

## Data Availability

Anonymised data collected for the study and a data dictionary defining each field in the set will be made available 1 year after publication, after approval of a proposal, and with a signed data access agreement. All data requests should be submitted to the corresponding author for consideration.

## Abbreviations

ATC: Anatomical Therapeutic Chemical classification system
CCM: Cough and cold medicines
CI: Confidence interval
ED: Emergency department
HER: Electronic health record
ENT: Ear, nose, and throat specialist
EMA: European Medicines Agency
FDA: Food and Drug Administration
GP: General practitioner
ICD: International Classification of Diseases
LRTI: Lower respiratory tract infection
PED: Paediatrician
URTI: Upper respiratory tract infection

## Code availability

Data available on request from the authors.

## References

[1] Thompson M, Vodicka TA, Blair PS (2013). Duration of symptoms of respiratory tract infections in children: systematic review. BMJ.

[2] Chang AB, Phelan PD, Sawyer SM, Robertson CF (1997). Airway hyperresponsiveness and cough-receptor sensitivity in children with recurrent cough. Am J Respir Crit Care Med.

[3] American Academy of Pediatrics (2013) Cough and cold medicines should not be prescribed, recommended or used for respiratory illnesses in young children. https://www.choosingwisely.org/clinician-lists/american-academy-pediatrics-cough-and-cold-medicines-for-children-under-four/. Accessed 7 Apr 2021

[4] Horton DB, Gerhard T, Strom BL (2019). Trends in cough and cold medicine recommendations for children in the United States, 2002–2015. JAMA Pediatr.

[5] US Food and Drug Administration (2018) Use caution when giving cough and cold products to kids. https://www.fda.gov/drugs/special-features/use-caution-when-giving-cough-and-cold-products-kids. Accessed 26 Mar 2021

[6] Health Canada (2008) Health Canada releases decision on the labelling of cough and cold products for children. https://www.healthycanadians.gc.ca/recall-alert-rappel-avis/hc-sc/2008/13267a-eng.php

[7] US Food and Drug Administration (2018) FDA requires labeling changes for prescription opioid cough and cold medicines to limit their use to adults 18 years and older. https://www.fda.gov/drugs/drug-safety-and-availability/fda-drug-safety-communication-fda-requires-labeling-changes-prescription-opioid-cough-and-cold. Accessed 7 Apr 2021

[8] European Medicines Agency (2015) Codeine-containing medicinal products for the treatment of cough or cold in paediatric patients. https://www.ema.europa.eu/en/medicines/human/referrals/codeine-containing-medicinal-products-treatment-cough-cold-paediatric-patients#overview-section. Accessed 28 Mar 2021

[9] Smith S, Schroeder K, Fahey T (2014). Over-the-counter (OTC) medications for acute cough in children and adults in community settings. Cochrane Database Syst Rev.

[10] Gardiner S, Chang A, Marchant J, Petsky H (2016). Codeine versus placebo for chronic cough in children (Review). Cochrane Database ofSystematic Rev.

[11] Morice AH, Millqvist E, Bieksiene K (2020). ERS guidelines on the diagnosis and treatment of chronic cough in adults and children. Eur Respir J.

[12] Hampton LM, Nguyen DB, Edwards JR, Budnitz DS (2013). Cough and cold medication adverse events after market withdrawal and labeling revision. Pediatrics.

[13] Kaiser SV, Asteria-Penaloza R, Vittinghoff E (2014). National patterns of codeine prescriptions for children in the emergency department. Pediatrics.

[14] Mazer-Amirshahi M, Rasooly I, Brooks G (2014). The impact of pediatric labeling changes on prescribing patterns of cough and cold medications. J Pediatr.

[15] Ingram J, Cabral C, Hay AD (2013). Parents’ information needs, self-efficacy and influences on consulting for childhood respiratory tract infections: a qualitative study. BMC Fam Pract.

[16] Stockwell MS, Catallozzi M, Larson E et al (2014) Effect of a URI-related educational intervention in early head start on ED visits. Pediatrics 133. 10.1542/peds.2013-2350 10.1542/peds.2013-2350 PMC4006431 24709931

[17] Grol R, Grimshaw J (2003). From best evidence to best practice: effective implementation of change in patients’ care. Lancet.

[18] Cabana MD, Rand CS, Powe NR (1999). Why don’t physicians follow clinical practice guidelines? A framework for improvement. JAMA.

[19] Lugtenberg M, Zegers-van Schaick JM, Westert GP, Burgers JS (2009). Why don’t physicians adhere to guideline recommendations in practice? An analysis of barriers among Dutch general practitioners. Implement Sci.

[20] The Finnish Medical Society Duodecim and the FPS and the FS of GMHTFMS (2014) Lower respiratory tract infections (children). Current Care Guidelines. In: Duodecim. www.kaypahoito.fi. Accessed 26 Mar 2021

[21] Kivistö JE, Poutanen R, Protudjer JLP (2021). Current Care Guidelines had no immediate effects on antitussive prescriptions to Finnish children. Acta Paediatr.

[22] Forrest D, Hoskins A, Hussey R (1996). Clinical guidelines and their implementation. Postgrad Med J.

[23] Palmu S, Mecklin M, Heikkilä P (2018). National treatment guidelines decreased the use of racemic adrenaline for bronchiolitis in four Finnish university hospitals. Acta Paediatr.

[24] Haskell L, Tavender EJ, Wilson CL (2021). Effectiveness of targeted interventions on treatment of infants with bronchiolitis. JAMA Pediatr.

[25] Diaz MCG, Handy LK, Crutchfield JH (2020). Impact of a personalized audit and feedback intervention on antibiotic prescribing practices for outpatient pediatric community-acquired pneumonia. Clin Pediatr (Phila).

[26] Rosenbluth G, Wilson SD, Maselli JH, Auerbach AD (2011). Analgesic prescribing practices can be improved by low-cost point-of-care decision support. J Pain Symptom Manage.

[27] Akenroye AT, Baskin MN, Samnaliev M, Stack AM (2014) Impact of a bronchiolitis guideline on ED resource use and cost: a segmented time-series analysis. Pediatrics 133. 10.1542/peds.2013-1991 10.1542/peds.2013-1991 24324000

[28] Bryan MA, Desai AD, Wilson L et al (2017) Association of bronchiolitis clinical pathway adherence with length of stay and costs Pediatrics 139. 10.1542/peds.2016-3432 10.1542/peds.2016-3432 28183732

[29] Kuitunen I, Artama M, Mäkelä L (2020). Effect of social distancing due to the COVID-19 pandemic on the incidence of viral respiratory tract infections in children in Finland during early 2020. Pediatr Infect Dis J.

