# Supplementary material for: Cough and cold medicine prescription rates can be significantly reduced by active intervention
Source: Eur J Pediatr. 2021 Dec 15;181(4):1531–9. doi: 10.1007/s00431-021-04344-0 (PMC8673918; doi:10.1007/s00431-021-04344-0)

Figure S1. Yearly cough and cold medicine prescriptions for three cough and cold medicine types classified according to the Finnish Medicines Agency classification (ATC, Anatomical Therapeutic Chemical classification system). Image created by the authors.

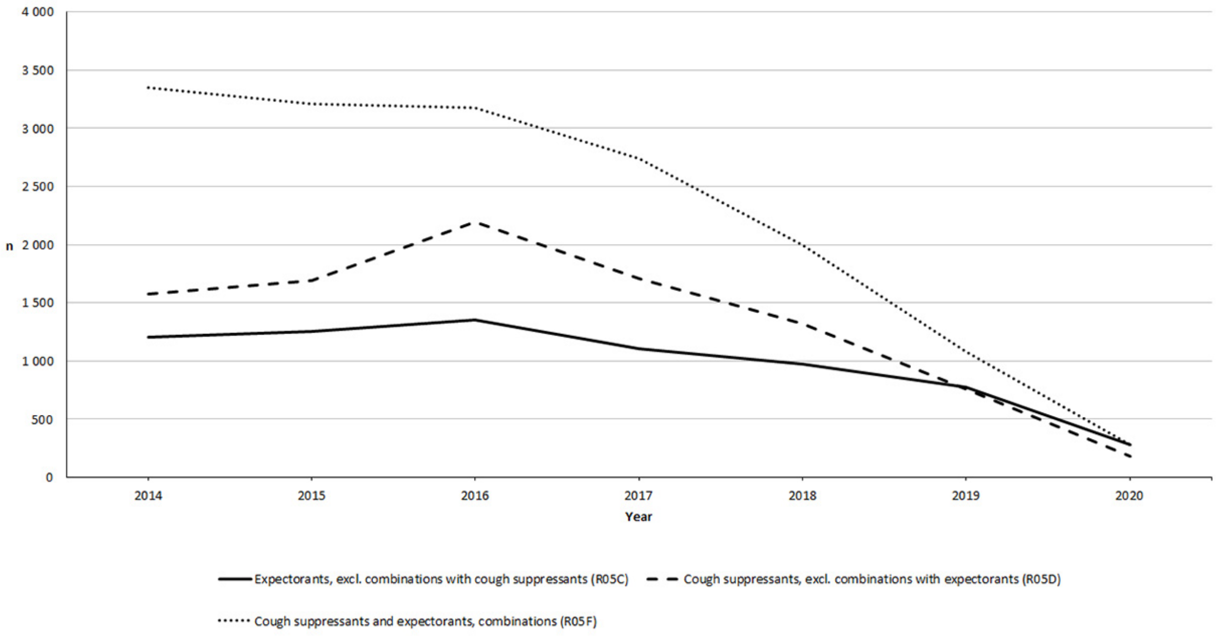

Supplement: Supplementary file 1 — Supplementary file1 (PDF 169 KB) [file 431_2021_4344_MOESM1_ESM.pdf]
