# Supplementary material for: Cough and cold medicine prescription rates can be significantly reduced by active intervention
Source: Eur J Pediatr. 2021 Dec 15;181(4):1531–9. doi: 10.1007/s00431-021-04344-0 (PMC8673918; doi:10.1007/s00431-021-04344-0)
Supplement: Supplementary file 2 — Supplementary file2 (PDF 86 KB) [file 431_2021_4344_MOESM2_ESM.pdf]

APPENDIX 1: ELECTRONIC HEALTH RECORD SYSTEM AND INTERVENTION MONITORING

DynamicHealth (TietoEVRY, Finland) is a tool utilised by Terveystalo for handling EHR data, such as patient personal information, diagnoses, prescriptions, laboratory values, referrals, performed procedures, and information about the practitioner. These records are stored on-premise in the Microsoft SQL Server database. The data for this analysis is collected and combined from tables containing the required personal information, visit information, diagnoses, and medications as they are registered in DynamicHealth by the practitioner. Patient age is derived from the birth date and defined as the patient's age during the visit in years. All patient data is actively managed and conforms to the European Union's General Data Protection Regulation (GDPR) and the data security legislation of Finland.

Each unit (clinic) has a chief physician, who has access to the clinical data and quality indicators from their own clinic using a dedicated dashboard. Terveystalo's chief medical officer and medical directors (i.e., head of paediatrics) can access the clinical data across the entire company. Throughout the active intervention period, this dashboard was used for monitoring prescription practices on an individual physician and patient level. The dashboard was built using the business intelligence tool QlikView (QlikTech International, Finland), which is used in Terveystalo to visualize data from various source systems. Terveystalo has been using QlikView since 2012, and the dashboard was built in 2018. The dashboard refreshes the data once per day from Terveystalo's Data Warehouse. The data warehouse utilizes automated data loading and updating processes. It also combines data from multiple databases, including DynamicHealth, once a day. QlikView end-user dashboards can be opened using a web browser or a desktop client with sufficient access rights.
