# Supplementary material for: Cough and cold medicine prescription rates can be significantly reduced by active intervention
Source: Eur J Pediatr. 2021 Dec 15;181(4):1531–9. doi: 10.1007/s00431-021-04344-0 (PMC8673918; doi:10.1007/s00431-021-04344-0)
Supplement: Supplementary file 3 — Supplementary file3 (PDF 87 KB) [file 431_2021_4344_MOESM3_ESM.pdf]

APPENDIX 2: COUGH AND COLD MEDICINE GROUPS

**Expectorants, excl. combinations with cough suppressants (R05C)**	**Cough suppressants, excl. combinations with expectorants (R06D)**	**Cough suppressant and expectorant, combinations (R05F)**	**Opioid derivatives, single or combinations**
Ambroxol<br>Ammonium chloride<br>Bromhexine<br>Carbocisteine<br>Erdosteine<br>Guaifenesin<br>N-acetylcysteine	Dextromethorphan<br>Ethylmorphine<br>Pentoxyverine<br>Codeine + ephedrine + coccillana	Cocillana<br>Codeine + guaifenesin<br>Codeine + guaifenesin + ammonium chloride<br>Codeine + diphenhydramine + ephedrine + cocillana<br>Dextromethorphan + ephedrine	Coccillana<br>Codeine<br>Dextromethorphan<br>Ethylmorphine
