# Supplementary material for: Cough and cold medicine prescription rates can be significantly reduced by active intervention
Source: Eur J Pediatr. 2021 Dec 15;181(4):1531–9. doi: 10.1007/s00431-021-04344-0 (PMC8673918; doi:10.1007/s00431-021-04344-0)
Supplement: Supplementary file 4 — Supplementary file4 (PDF 78 KB) [file 431_2021_4344_MOESM4_ESM.pdf]

APPENDIX 3: INTERNATIONAL CLASSIFICATION OF DISEASES 10 USED IN THE STUDY

The International Classification of Diseases 10 (ICD-10 codes) for LRTI included J09, J10, J10.0, J10.1, J10.8, J11, J11.0, J11.1, J11.8, J18, J18.0, J18.1, J18.8, J18.9, J20, J20.0, J20.3, J20.4, J20.5, J20.6, J20.8, J20.9, J21.90, J21.99, J21.8, J21.0, J21, J22, and J21.9. Cough was defined by ICD-10 code R05, and URTI was defined by codes J06, J06.80, J06.89, and J06.9.
