# Supplementary material for: Cough and cold medicine prescription rates can be significantly reduced by active intervention
Source: Eur J Pediatr. 2021 Dec 15;181(4):1531–9. doi: 10.1007/s00431-021-04344-0 (PMC8673918; doi:10.1007/s00431-021-04344-0)
Supplement: Supplementary file 5 — Supplementary file5 (PDF 107 KB) [file 431_2021_4344_MOESM5_ESM.pdf]

APPENDIX 4: INITIAL ASSESSMENT AND STEPS BEFORE THE ACTIVE INTERVENTION

**Initial assessment**	2016 DEC.	• Assessing the rate of CCM prescriptions.<br>• Defining the problem.
**Initial steps to increase physicians' knowledge before active intervention**	2017 JAN.–DEC.	• Standard dissemination of guidelines (e.g., reminders about guidelines via e-mail and in the company's intranet).<br>• Talking about the guidelines in regular educational meetings.
**Assessment before active intervention, intervention objectives, and evaluation plan**	2018 JAN.–FEB.	• Determining the baseline parameters before intervention.<br>• Identification of target groups, stakeholders, and key determinants.<br>• Setting intervention goals (i.e., zero CCM prescriptions by the end of 2020), indicators, and measures.
**Intervention strategies**	2018 JAN.–FEB.	• Defining intervention steps and selection of methods and strategies.<br>• Development of practical materials (e.g., short and easily accessible version of guidelines and patient education material).
**Intervention and evaluation plans**	2018 JAN.–FEB.	• Defining the objectives and metrics.<br>• Identifying the need for an effective, electronic nationwide monitoring tool (to be tailored for the intervention).<br>• Specifying the active intervention period (from MAR. 2018 to DEC. 2020).

CCM, cough and cold medicine
