# Supplementary material for: Cough and cold medicine prescription rates can be significantly reduced by active intervention
Source: Eur J Pediatr. 2021 Dec 15;181(4):1531–9. doi: 10.1007/s00431-021-04344-0 (PMC8673918; doi:10.1007/s00431-021-04344-0)
Supplement: Supplementary file 6 — Supplementary file6 (PDF 131 KB) [file 431_2021_4344_MOESM6_ESM.pdf]

APPENDIX 5: TIMELINE OF INTERVENTION IMPLEMENTATION (ACTIVE INTERVENTION PERIOD)

**Step 1**	2018 MAR.	• Objectives of the intervention were published and disseminated via multiple channels (material in print, online, e-mails, unit meetings).<br>• Press release (for laypeople) about the intervention.<br>• Concise, practical written material distributed for physicians and caregivers (also easily accessible online).<br>• ***Real-time nationwide evaluation tool (dashboard) based on EHR data made available for monitoring the progress of the intervention.***
	2018 JUN.	• Progress report and general positive feedback.
	2018 AUG.–OCT.	• Educational meetings held by opinion leaders.
	2018 OCT.	• Progress report and constructive feedback.
**Step 2**	2019 JAN.<br>2019 FEB.<br>2019 MAR.<br>2019 MAR.–JUN.	• Progress report and feedback.<br>• Reminder letters to all physicians and unit* leaders.<br>• Status report for each unit and specialty† group.<br>• Identification of noncompliant units and physicians.<br>• Educational meetings held by opinion leaders‡.<br>• Meetings with chief physicians of noncompliant units. Focus meetings in selected units. Outreach visits. Individual feedback to noncompliant physicians.
**Step 3**	2019 SEP.<br><br><br><br><br><br>2019 DEC.<br>2020 JAN.–MAR.<br>2020 SEP.<br>2020 SEP.–OCT.<br>2020 DEC.	• Status report by individual physicians.<br>• Status report and thank you letter to everyone about progress.<br>• Individual feedback to top 50 noncompliant (most actively prescribing) physicians.<br>• Progress report.<br>• Personalized letter and phone calls to those physicians who were actively recommending CCM.<br>• Progress report.<br>• Final contact to those physicians who were still actively recommending CCM.<br>• End of active intervention.
**Intervention evaluation**	2021 FEB.	• Evaluation according to predefined metrics.

*Terveystalo has over 300 units nationwide.
†Over 50 specialties.
‡Opinion leaders and experts from within the company and invited speakers.
CCM, cough and cold medicine. EHR: Electronic health record.
