# Supplementary material for: Cough and cold medicine prescription rates can be significantly reduced by active intervention
Source: Eur J Pediatr. 2021 Dec 15;181(4):1531–9. doi: 10.1007/s00431-021-04344-0 (PMC8673918; doi:10.1007/s00431-021-04344-0)
Supplement: Supplementary file 7 — Supplementary file7 (PDF 80 KB) [file 431_2021_4344_MOESM7_ESM.pdf]

## APPENDIX 6: SUPPORT MATERIALS FOR THE INTERVENTION

At the beginning of the intervention and each year thereafter, we produced a press release directed to the public about the current recommendations and about our intervention study. CCM prescription rates were also published in the company's annual quality reports, which were freely available on the company's website and distributed in print to all stakeholders and interest groups.

Concise, practical materials were written, which included information on the Finnish Current Care Guidelines and on the ineffectiveness and potential harmfulness of CCMs. Separate material was distributed to physicians and caregivers and was made easily accessible online in intranet and extranet.

Instead of prescribing CCMs, physicians were encouraged to give the families a ready-made written information package. This included information about the nature of paediatric coughs, recommended treatment options, possible side effects of CCMs, and instructions when additional medical help, such as a doctoral visit, would be needed. There were also regular progress reports and positive feedback letters to unit chief physicians and individual doctors.
