# Supplementary material for: Cough and cold medicine prescription rates can be significantly reduced by active intervention
Source: Eur J Pediatr. 2021 Dec 15;181(4):1531–9. doi: 10.1007/s00431-021-04344-0 (PMC8673918; doi:10.1007/s00431-021-04344-0)
Supplement: Supplementary file 8 — Supplementary file8 (PDF 81 KB) [file 431_2021_4344_MOESM8_ESM.pdf]

APPENDIX 7: COST CALCULATIONS

Intervention costs were estimated by multiplying the work hours used for the intervention by mean salaries or missed mean invoicing of participants. First, the work hours used in every professional group were estimated. Then, the current mean salaries provided by each trade union were used in calculations. For intervention developers, the mean salaries of €65.35/hour and €51.04/hour were used, depending on the professional position. Additionally, the mean salary of €20.95/hour was used for the communicating work and €26.15/hour for data management. The missed mean invoicing of €183/hour and €267/hour was used for the general practitioners (GPs) and paediatricians or other specialists, respectively. Both working hours and salaries were estimated separately for each intervention step. The employers' side expenditures related to salaries were not considered.
